# Intraspecific variability of social structure and linked foraging behavior in females of a widespread bat species (*Phyllostomus hastatus*)

**DOI:** 10.1371/journal.pone.0313782

**Published:** 2025-03-20

**Authors:** María C. Calderón-Capote, M. Teague O’Mara, Margaret C. Crofoot, Dina K.N. Dechmann

**Affiliations:** 1 Max Planck Institute of Animal Behavior, Radolfzell, Germany; 2 Department of Biology, University of Konstanz, Konstanz, Germany; 3 Smithsonian Tropical Research Institute, Gamboa, Panamá; 4 Department of Biological Sciences, Southeastern Louisiana University, Hammond, Louisiana, USA; 5 Bat Conservation International, Austin, Texas, USA; 6 Cluster for the Advanced Study of Collective Behavior, University of Konstanz, Konstanz, Germany; National Museums of Kenya, KENYA

## Abstract

Intraspecific variation in morphology and behavior is widespread, especially in species with large distribution ranges. This includes foraging which can vary according to the local resource landscape. How this may be linked to differences in social structure, especially in socially foraging species is less known. Greater spear-nosed bats are well known for their large repertoire of often highly complex social behaviors. In Trinidad, they form stable groups of unrelated females that recruit other members to temporally unpredictable flowering balsa trees. We compared these findings with a dataset of capture data, GPS tracks, and observations collected over six years in a colony in Panamá. We found profound differences in the foraging behavior and group stability of *Phyllostomus hastatus* during the dry season where social behaviors were expected. Female bats did not coordinate commutes to exploit distinct foraging resources as a group. Instead, females commuted individually to very distant foraging areas which overlapped between groups. Linked to this we found groups to be unstable in size over the short and long term. Our findings highlight the large intraspecific variation and indicate a strong influence of the local resource landscape and associated benefits of social foraging on the social structure in these bats and possibly many other animals.

## Introduction

Spatiotemporal variation in the distribution and abundance of resources can drive intraspecific variation in behavior, especially foraging, of species, ultimately affecting fitness [1,2]. Divergent foraging strategies can result from the effect of different local conditions on the distribution of crucial resources or competitive regimes [3–6]. For instance, rockhopper penguins (*Eudyptes chrysocome*) exhibit flexible foraging strategies depending on the local prey availability [7]. In areas with less prey, these penguins have shorter dive durations, and are consequently less active, which negatively affects chick growth [7]. Australasian gannets (*Morus serrator*) forage at different water depths depending on prey availability [8]. Similarly, grey seals (*Halichoerus grypus*) also alter their foraging efforts seasonally, increasing activity during winter when prey migrate or move to deeper waters and decreasing it during summer as prey move to shallower areas [9]. These differences are not only important for understanding the role of these predators in the local ecosystems but can also be indicative of intraspecific flexibility under changing conditions.

While the link between foraging behavior and resource variation has been widely studied [6,10], it has also been long recognized that the environment can shape the social structure of animals [11]. However, to what degree intraspecific variation in social systems is evolved and thus adaptive or based on inherent flexibility is still poorly understood. Studies of populations in species with large distribution ranges living in variable (i.e., seasonal) environments allow comparisons that may help elucidate these non-exclusive scenarios. Socioecological models suggest that predation risk forces species to form groups [12], but the strength of those relationships will be determined by competition for resources [13–16]. In cases when resources are unevenly distributed in space and time and competition is high, groups will tend to form long-lasting relationships to benefit from the defense of high-quality resources [11, 13, 17]. How animals adjust their behavior in response to others and the environment is essential to understanding species’ flexibility and long-term behavioral patterns in wide-ranging species [3,4,18,19].

A common strategy to improve foraging decisions in unpredictable food landscapes is the use of social information [6,20,21]. Food availability can be unpredictable at the broad and fine-scale in both space and time [6] and social information from group members can increase foraging efficiency and success but potentially increases competition [22–24]. For example, wild chimpanzees (*Pan troglodytes*) are known to recruit group members to fruit trees, which are difficult to find, but offer abundant food which remains available long enough to share information about it [25]. Chimpanzees even modify call structure to convey information about the species and abundance of fruit [26]. This and similar behaviors allow animals to exploit the knowledge and experiences of others, reducing the risks associated with trial-and-error learning [27]. Whether and how social information is used is the result of an intricate interplay of current distribution and abundance of food, an individual’s energetic need and the social environment [28,29]. For example males of some temperate zone bat species successfully forage on swarming insects alone most of the year, but when they face the increased energetic costs of sperm productions they temporarily form colonies to profit from eavesdropping on the foraging success of others [28–30]. As this example shows, social information can be particularly advantageous at certain points of an animal’s life or in a certain environment. However, using social information is not the only effective strategy. Personal information derived from past experiences can also allow individuals to effectively find and exploit available feeding resources [31]. Frugivorous animals are known to use long-term memory to remember and monitor fruiting trees and additionally, they have goal-oriented trajectories to reach frequently used food patches [31,32]. This might facilitate foraging by reducing the cognitive effort of spatial navigation [33]. Thus, the decision of whether to use social or individual foraging might depend on the temporal and spatial variability in the local resource landscape and may allow populations to retain effective foraging strategies that enhance overall adaptability and survival [6].

The greater spear-nosed bat, *Phyllostomus hastatus*, is highly social. In Trinidad, *P. hastatus* form stable long-term groups of unrelated females [34]. These groups coordinate reproduction and show many complex social behaviors including guarding each other’s pups [35]. The fact that group members invest in each other’s fitness might explain the extended benefits of foraging in groups under resource unpredictability [34–36]. During the dry season when bats transition from an omnivorous to a more pollen/nectar-based diet [36], females of *P. hastatus* use group distinctive calls – known as screech calls – to coordinate foraging movements and recruit other group members to feed on and defend unpredictable flowers of balsa trees [36–38]. However, in a geographically distant population in Bocas del Toro, Panamá, these patterns were not confirmed [39]. Female group members did not coordinate foraging movements, but rested together near the foraging areas [39]. This implies clear behavioral differences between these two regional populations, and that such variations in yearly or seasonal foraging strategies of *P. hastatus* across their geographical range can be a significant pointer to the important role of local or regional habitat variables and conditions in driving the social structure of a widely-ranging species

We used miniaturized GPS devices to track foraging movements of groups of females of *P. hastatus* during the dry season of 2022 in Bocas del Toro, Panamá. We hypothesized that female groups would engage in social foraging and form stable groups during the dry season when their most consumed resource, balsa flowers, were unevenly distributed and temporally unpredictable in the landscape. We expected female group members to coordinate departures and returns from the roost to the foraging areas. We also predict that if bats follow group members to foraging patches, commuting paths should be more similar to those of group members than those of other groups. Additionally, if group members defend balsa trees as described from Trinidad, we expected social groups to have shared foraging areas that differed from those of other groups [36]. We also compared group-level summary statistics with previously collected data from 2016 in the same colony, to investigate long-term behavioral patterns in the dry season, and strengthen the validity of our observations. Finally, we compare our results to the behavior of the *P. hastatus* Trinidad population [34,36], to better understand the extent of intraspecific variation in sociality among *P. hastatus* females.

## Materials and methods

### Ethics

This study was conducted under the permit of Ministerio del Ambiente, Panamá (SE/A-96-15, SE/A-96-18, SE/A-38-2020), and the Animal Care and Use Committee at the Smithsonian Tropical Research Institute (2014-0701-2017, 2017-0815-2020-A2, 2020-0212-2023), and adhered to the ASAB/ABS Guidelines for the Use of Animals in Research.

### Capture and tracking movements

We captured 61 *P. hastatus* females in the “la Gruta” colony in Isla Colón, Bocas del Toro, Panamá, during the dry season in February and March of 2016 (n =  35) and 2022 (n =  26). Isla Colón is the largest main island of the Bocas del Toro Archipelago and the most densely-populated by humans. The Island, as opposed to other parts of Panamá, has a Caribbean seasonal pattern, with a dry period in February and March and a second one, in September and October. The wet season has two peaks in rainfall during July and December [40].

We caught bats using either a bucket trap or a ring-shaped trap with a net that was placed directly over the cavities where social groups roosted. We captured most bats in the groups, but occasionally, one or two bats managed to escape out of the trap. Groups usually consisted of one dominant male, several females and sometimes one or two subdominant males. Bats were transferred into soft cloth bags, processed immediately and released within 3 h at the capture site. We measured bats’ forearm length ( ± 0.01 mm), mass ( ± 0.5 g), marked them with subcutaneous PIT tags (ID 100 Transponder, Trovan^®^) and determined their sex, reproductive status and age (juvenile or adult). Only adult female bats were tracked using biologgers wrapped in shrink tubes to avoid humidity damaging the device. We glued tags to the bats’ dorsal fur below the shoulder blades (Osto-bond skin bonding latex adhesive, Montreal Ostomy). Females weighed 118.1 ±  7.35 g, the biologgers weighed 6.44 ±  0.48 g which represented 5.46 ±  0.46% of their total body mass (S1 Table).

Across years we tracked bats with tags of different vendors with different programming schedules. In 2016, tags (Gypsy-5 GPS, TechnoSmArt) collected GPS locations every 1 or 2 s (see [39] for details). When adequate GPS reception was not available, tags went into a low energy sleep state for 5 min and then restarted to search for satellites for 90 s. Performance of the GPS tags was variable because of the depth of the roosting cave and the density of foliage where the bats foraged. In February 2022 tags (Vesper, ASD) took GPS fixes every 2 min. All tags collected data from 18h–06 h local time. We included only data from individuals that had at least one night of tracking with complete in- and outbound commutes. All data was downsampled to two-minute intervals to make analysis comparable.

### Behavioral classification

We fitted a three-state hidden Markov model (HMM) for each bat night using the momentuHMM package to identify behaviors [41], assuming that movement could be classified into three biologically meaningful behaviors for the species [39]. To implement the HMM we first regularized the tracks by inserting “NA” for missing observations to obtain a complete series of two minute intervals, using the setNA function from the adehabitatLT package [42]. A previous study found social resting between foraging as an important behavior [39]. However, after down-sampling the data resolution did not accurately distinguish between the categories used there (slow/fast foraging and resting). Thus, we fitted a two-state model with “foraging” (short movements with low persistence of direction including potential resting) and “commuting” (fast and directed movement) as categories even though three-state models had lower AICs. The model was fitted using step lengths (assuming states could be described using a mixture of Gamma distributions), and turning angles, with wrapped Cauchy distributions [41]. Behavioral categories were also corroborated by visual inspection after the classification.

### Group stability

We visually counted the number of bats in social groups once a week starting in October 2021 (end of wet season) until January 2022 (start of the dry season) to assess group stability. We took high resolution pictures of the groups and counted individual bats. When we captured a group for tracking purposes, we recorded this disturbance and the number of recaptured bats. We also include the size of the groups that were tracked in 2016, however these counts were only performed once.

### Foraging coordination

#### Departure and return coordination.

To assess coordination in departures and returns we calculated pairwise time difference by night between individuals of the same and different groups in 2022. We extracted departure and return times for each individual bat night from tracks where the complete night had been recorded. Departures corresponded to the first GPS fix once the bat left the cave, and returns corresponded to the last GPS fix before the bat entered the cave after commuting back. We grouped differences in departure and return times in binary variables “departure together” or “return together” to note when pairs of bats could have left or arrived together or not. We set the threshold for pairs of bats departing or returning together to two minutes, which matched the resolution of the GPS fixes. This also avoided large time gaps where bats could not have maintained contact (hearing distance 290 m, [39,43]). Although bats can fly a large distance (around 1 km) in a straight line at an average ground speed of 8.63 m/s in two minutes [39], we assumed that they might have used this time to acquire social information from others. This could occur if they did not fly directly towards the foraging area, but swarmed around the cave. As very few bats departed together, we then explore the general differences in departure and return times within individuals of the same and different groups with a generalized linear mixed effect model (GLMM) with a gamma distribution [44]. We used “group” as a fixed effect and bat dyads and date as a random effect.

#### Commuting path coordination.

We investigated trajectory similarity between pairs of individuals from the two groups tracked during the dry season of 2022 by using only nightly outbound commutes. We expected outbound trajectories to be more coordinated (similar to each other) assuming bats would fly to balsa trees together. To calculate trajectory similarity, we use dynamic time warping (DTW) algorithm, which finds the optimal alignment (smallest normalized distance) between each pair of time series [45]. For the DTW function, we used UTM coordinates of the path trajectories as the input variables using the Euclidean distance method. DTW is a useful measure because it reduces distortion and shifting by allowing an elastic transformation of the time series to detect similar shapes even in trajectories with different lengths [45]. DTW however, can be sensitive to noise as all points in the trajectory are matched, including any outliers. To test if trajectories were more similar among individuals in a group than between groups we modeled these normalized similarity distances using a linear mixed model assuming a lognormal distribution after checking the distribution of the response variable [44]. We used the log transformed similarity distance between pairs of trajectories as a response variable, group identity as an explanatory variable, and bat dyads as random effect. We evaluated significance of the fixed effects by calculating type III p-value with the Satterthwaite’s method using anova [46].

### Foraging area overlap

We calculated foraging area overlap by estimating Autocorrelated Kernel Density Estimates (AKDE) from individual foraging points using the ctmm package [47]. We used foraging locations with at least 10 relocations where bats stayed more than 8 mins. We excluded foraging locations on Isla Colón (roosting island), because the main foraging areas were located outside their roosting island between 15–25 km away (S1–S2 Fig [48]). We used a grid cell size of 500 m and ensured that the extent of the grid will cover the AKDE estimation. We used the 95% contours of the AKDE to extract overlap between individuals from the same group and from different groups in the dry season of 2022. We use the Bhattacharyya similarity coefficient (BA) from the function overlap in the ctmm package to quantify similarity (overlap) between the density distribution of the AKDEs [49]. This function calculates the ratio of the intersection area to the average individual area and returns values between 0 (no overlap) and 1 (full overlap) [49]. To understand if AKDEs within individuals from a group were more similar to each other than between groups we used a GLMM with a beta distribution with a probit function [50]. Beta distribution is appropriate to use for response variables bounded between 0 and 1 [51]. We used the overlap value as a response variable, a binary variable describing if the individuals belong to the same group or not as an explanatory variable and bat dyads as random effects.

### General activity budgets

We calculated the proportion of time spent in each behavior for each bat night, in each year when possible. We used a binomial GLMM to test if commuting and foraging differed between years. We used proportion of time as response variable and year as an explanatory variable. We removed identity from the random effects as there was little variation explained by it.

In all GLM models used in this study, significance was assessed at p-value threshold of ≤  0.05. We verified that the data met the model’s assumptions by assessing normality and dispersion using the DHARMa package [52]. All analysis was conducted in R 4.3.3 [53].

### Social vocalizations

We inspected data from two of the bats tagged with audio recorders during the dry season of 2016 for the presence of screech calls. One bat was recorded for 4.5 h and the other during 3 h while foraging.

## Results

Overall, we analyzed data of four female groups from the end of 2021 to the beginning of 2022 to assess group stability, and GPS data of 25 females (2016: n =  13, 2022: n =  12 [group F1 =  4, group F2 =  8], S1 Table) to test for coordination in departures, returns, commuting paths, and foraging area overlap.

### Group stability

**Fig 1:**
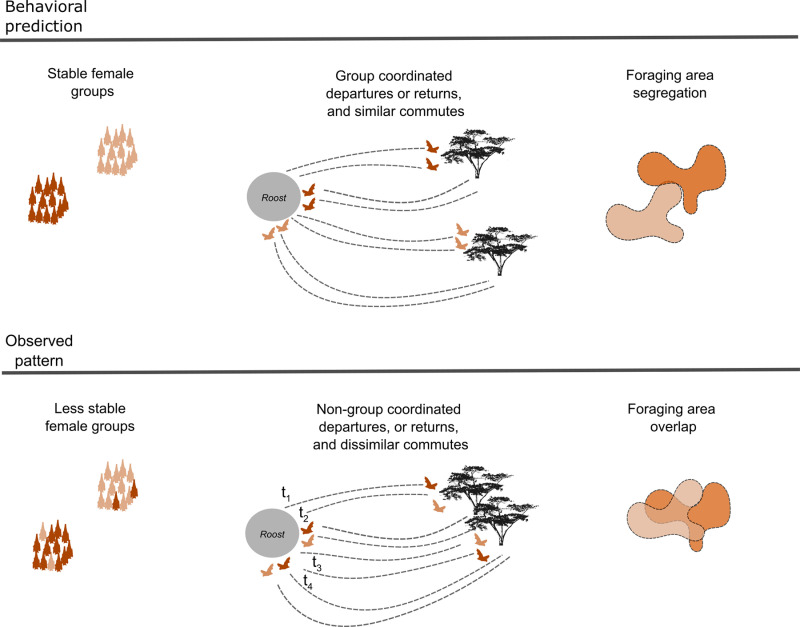
Foraging predictions based on the *P. hastatus* Trinidad population and observed foraging patterns in Bocas del Toro. Top panel: In Trinidad female groups of *P. hastatus* are stable for long periods (years). Female groups coordinate foraging movements by using screech calls during the dry season, especially during departures. Similarly, they should present higher path similarity within than between groups (not tested in Trinidad, but assumed as part of recruitment behavior). Additionally, foraging areas of social groups are spatially segregated, following the resource defense hypothesis [36]. Bottom panel: In our population in Bocas del Toro, groups of females were less stable, and females even joined other groups especially when disturbed (Fig 2C). Female groups did not coordinate departures, returns, or commutes, but foraging areas nonetheless overlapped (i.e., they foraged in the same areas but did not travel to them together).

**Fig 2:**
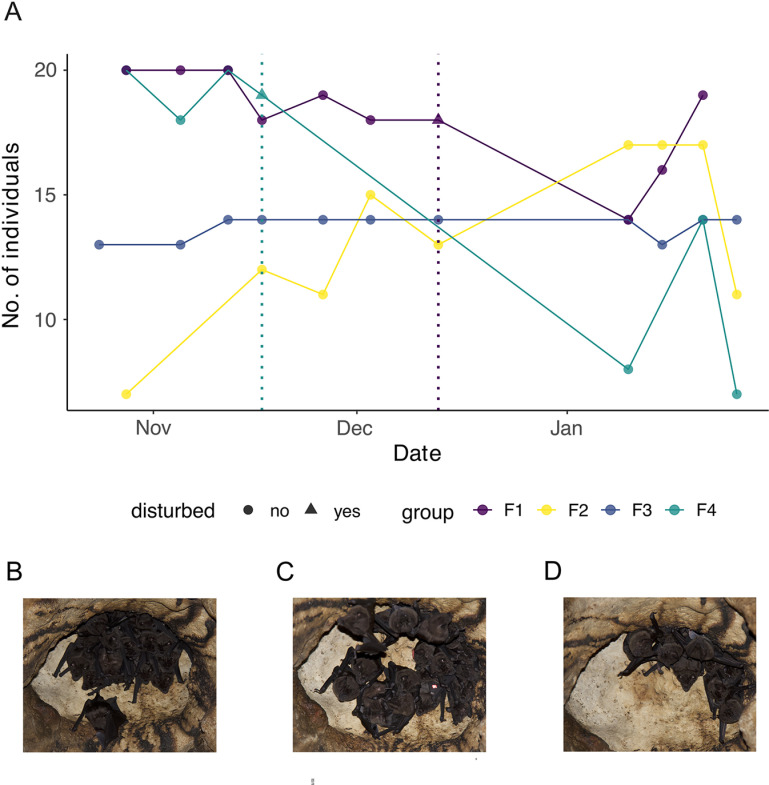
Weekly counts of the female groups of *P. hastatus* in La Gruta during the dry season of 2022. (A) Stability of female groups during late wet season and early dry season of 2022. Triangles and vertical lines represent a capture event, where bats were disturbed. After the capture events groups split or individuals in the group joined other existing groups. The initial individuals in the group did not reunite either in other locations in the cave or in the initial roosting hole. Example of group size changes of group F1 (purple line in the graph): (B) Initial group size, (C) Group of similar size, but containing individuals from other groups (a bat marked with reflective tape does not belong to this group). (D) After disturbance the group reduces in size.

We found that when groups were not disturbed they remained relatively stable in size over time (Fig 1–2, group F3). However, stability in group size varied during the three months monitored, especially after capturing events (Fig 2A, group F1, F2, F4). In 2016 we observed group sizes of 11, 6 and 22. No regular counts were done but tagged bats did not come together as a group after capture and recaptures from 2016 to 2022 were low.

### Foraging coordination

#### Departure and return coordination.

Female bats did not consistently depart immediately after sunset; rather, departure times varied over 147.99 ±  39.11 min after sunset in 2016 and 185.97 ±  104.06 min after-sunset in 2022 (S1 Fig).

**Fig 3:**
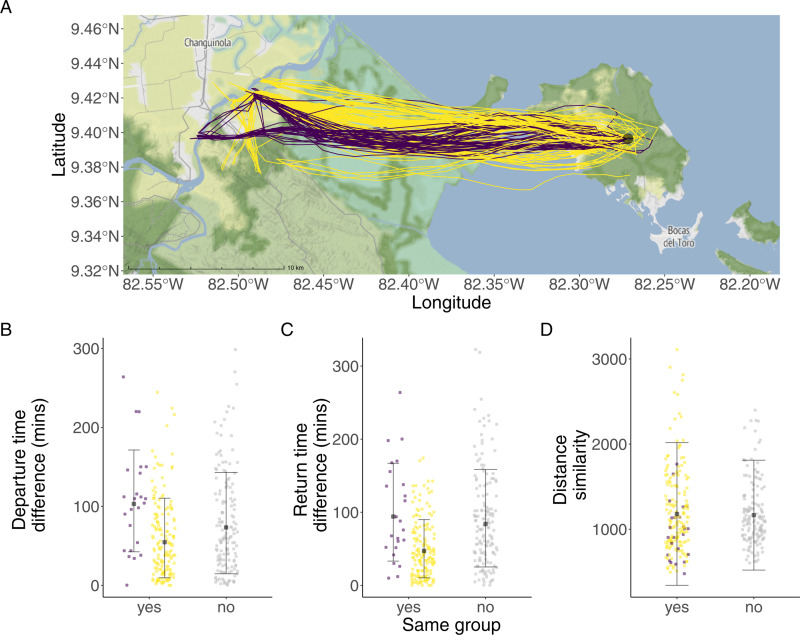
Group members did not coordinate departures, returns or commutes. (A) Movement trajectories of group F1 (purple) and F2 (yellow) who followed similar routes independent of group identity. Bats traveled from Isla Colón (circle represents La Gruta Cave) to the mainland in Changuinola. (B) Bats from the same or different groups did not depart together. (C) Bats from the same or different groups did not return together. (D) Bats had similar commuting paths (based on DTW normalized distance similarity values) independent of the group membership. Grey points: bats from different groups. Error bars in black represent the mean and standard deviation of the predicted values from the models. “Top map: reprinted from Stadiamaps under a CC BY-NC-SA 4.0 license, with permission from Stamen MapLibre styles, original copyright 2024”.

From 326 pairwise comparisons only 1.8 and 1.5% corresponded to dyads departing and returning together respectively (Fig 1). Departure time difference within and between groups was dissimilar (reference group F1 =  100.70 min (intercept), between groups F1F2 =  71.47 min, p =  0.22, group F2 =  51.08 min, p =  0.02, Fig 3A). Similar, was the return time difference within and between groups (reference group F1 =  94.42 (intercept), between groups F1F2 =  72.31, p = 0.37, group F2 =  43.68, p =  0.01; Fig 3B). Similar patterns were observed in 2016. Bats did not coordinate departure (54.14 ±  42.43 min) or returns (58.62 ±  48.39 min, n =  13).

#### Commuting path coordination.

Bats spent similar amounts of time commuting in 2016 (88.84 ±  16.55 min per night; n =  13) and in 2022 (112.48 ±  16.39 min; n =  12; intercept =  ‒0.22, estimate =  0.04, p-value =  0.93, S2 Fig). Groups did not follow distinct routes that differed from other groups. The DTW distance similarity values of commuting paths within and between groups in 2022 did not differ (intercept =  1215.01, estimate =  1133.42, p-value =  0.45, Fig 1 and 3D).

#### Foraging area overlap.

**Fig 4:**
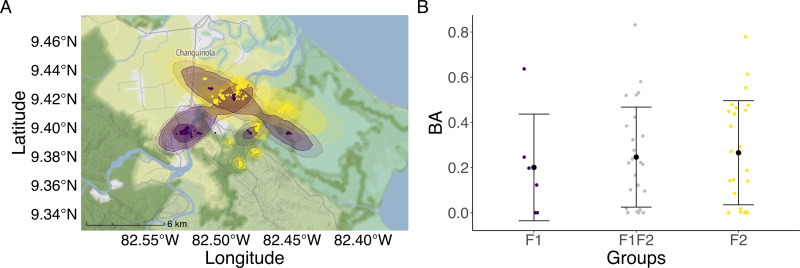
Foraging area overlap of female groups F1 (purple) and F2 (yellow). (A) AKDEs of female groups in their main foraging area (mainland in Changuinola). (B) Overlap (Bhattacharyya coefficient - BA) of foraging areas within (F1/F2) and between groups (F1F2, grey color) was similar. Error bars represent the mean and standard deviations. “Reprinted from Stadiamaps under a CC BY-NC-SA 4.0 license, with permission from Stamen MapLibre styles, original copyright 2024”.

In 2016 bats foraged for 121.05 ±  47.08 min each night (n =  13), and in 2022 for 150.58 ±  70.4 min (n =  11; intercept =  0.22, estimate =  ‒0.02, p-value =  0.96, S2 Fig). On average, individual foraging areas overlapped spatially by 20% both within and between groups (intercept =  0.19, estimate =  0.21, p-value =  0.74), and there was no distinct segregation in the foraging areas between members of the group, indicating overlap (Fig 4).

### Social vocalizations

After inspecting audio recordings from two of the female bats in 2016, we did not detect screech calls while flying or at their foraging areas.

## Discussion

Our study shows that greater spear-nosed bats have high intraspecific variation not only in their foraging behavior, but also in social structure in two distant populations. Compared to the well-studied Trinidad population we found less stable female groups and no coordination of foraging movements to preferred areas during the dry season. However, foraging areas showed a high degree of overlap between groups. We found no evidence for the use of screech calls to coordinate movements and defend high quality but temporally unpredictable resources (Fig 1). Thus, our data do not support the hypothesis that resource defense is one of the reasons for the formation of female groups at least in La Gruta as has been postulated for the Trinidad population. But, the fact that individuals used similar areas which were located at similar extremely long distances from the roost (around 25 km, twice the maximum distance in Trinidad), suggests that females may rely on social information from others at least to initially find abundant patches of balsa trees [39].

In this study, we captured bats using bucket or ring traps, following a methodology similar to that used in the Trinidad population [34]. While this capture method can temporarily disrupt grouping patterns – causing individuals to shuttle between female groups or split or switch their original roosting locations – the Trinidad female groups often managed to regroup in new roosts within weeks after capture, remaining relative stable over the years [34,35]. In contrast, female groups in our Panama population did not show the same resilience; they split up and were not observed to reunite, either in their original roosts or elsewhere within the cave. The cohesiveness displayed by the Trinidad bats, despite disturbances, suggests that Panama’s female groups may be inherently less cohesive and more susceptible to disturbance. However, further research is needed to better understand the extent and impact of our capture methods on their social dynamics.

From data collected six years earlier in the same colony in Bocas del Toro, we knew that female groups in 2016 did not coordinate foraging movements [39]. However, we assumed that 2016 was an usual year, due to low and late balsa flowering [54]. We thus expected a more similar pattern to Trinidad during our study six years later with female groups coordinating departures/returns, commutes and foraging areas. To our surprise foraging movements and use of the landscape continued to be similar in 2022. Several reasons can explain the present patterns: First, bats do not form as closed and stable groups as the female groups in Trinidad, and this flexibility in social structure may be altering the strength in which cooperative behaviors appear. La Gruta *P. hastatus* are 10–20% larger than those in Trinidad [55]. However, while body size is known to be correlated with foraging distance, possibly at least partially explaining the much longer commutes, this should not affect social foraging behavior [56,57]. Third, it is possible that bats use memory of prior experiences rather than exclusively social information to navigate the landscape, based on the directed movements and repeated use of foraging areas observed (Fig 3 and 4). Many animals use memory during foraging [58,59] including other bats [60–62]. However, if they use social information from others, benefits seem larger when the knowledge of the landscape is poor, i.e., at their early stages of life, or when there is a drastic change in resources due to climatic conditions, such as the onset of balsa flowering in the dry season. Thus, bats could initially benefit from information from group members and get informed about the current food resources available. Once a bat has learned of an available foraging patch/patches, individuals do not need to coordinate foraging movements with others. Similar patterns of food finding are observed in young gannets (*Morrus serrator*) that learn foraging paths from adults [8] or in pups of Egyptian fruit bats (*Rousettus aegyptiacus*) that learn initial foraging trees and corresponding commuting routes from their mothers [63]. Fourth, other factors such as topography, type of vegetation and changes in land cover due to anthropogenic disturbance might also play a role in the differential foraging patterns found between the two island populations. While both islands have relatively similar climatic conditions characteristic of tropical climate [40,55], Trinidad is a mountainous region with abundant tropical humid vegetation [55]. On the contrary, Bocas del Toro Archipelago has a vegetation characteristic of low coastal areas which has suffered land cover modifications since the 20^th^ century due to extensive banana plantations [64]. These different biogeographical patterns may have an impact on the distribution and abundance of local resources in both islands resulting in the different foraging strategies observed in both populations. For example, it is possible that the distribution and abundance of balsa trees on the mainland in Panama (not measured in this study) is higher and more predictable than in other parts of the landscape, i.e., inside the island, during the dry season. Thus, sharing foraging areas with several individuals of the cave without apparent intraspecific competition would pay the effort of flying such long distances. Ground truthing of tracking data corroborated the abundant yet spatially dispersed distribution of balsa trees during the dry season, suggesting an altered ecological context that could drive individual foraging. Overall, differences in group structure and changes in local conditions in terms of resource landscape could strongly explain the highly dissimilar social behaviors between years and populations.

We observed additional differences in our data compared to Trinidad. In Trinidad, bats departed within 30 min of each other to forage and returned within 2 h of being outside [34,36], whereas timing of departure in our La Gruta colony bats was irregular night by night and bats returned to the roost only after 3–4.5 h of foraging (S1–S2 Fig). These irregular departure timings conflict with what is known of the balsa flowers in Panamá [54]. Balsa has the highest nectar production in the early evening, declining overnight. Additionally, balsa flowers are a food source to many other vertebrate species such as oropendolas, kinkajous or capuchin monkeys [54], which feed just before or just after sunset, thus implying that the main competition for resources is not just other bats. Nonetheless, we would have expected females to regularly depart right after sunset to take advantage of higher amounts of sugar and nectar given their long commutes. That bats did not depart right after sunset indicates that other mechanisms rather than food competition are driving the timing of their foraging patterns.

Given the lack of female coordination of foraging movements in 2016 and 2022, it was not surprising that females commuted individually instead of with group members. The commuting paths of *P. hastatus* are mostly straight flights with narrow turning angles to exploit consistent foraging areas every night [48]. This strategy can be an efficient way to exploit the landscape by using goal-oriented paths rather than following other group members’ routes. This reinforces the idea of the bats’ ability to use memory to locate food resources. These patterns are similar to other frugivorous animals, such as primates [31,58] and bats [32,61,62], which integrate map-based navigation to access preferred foraging locations. More complex cognitive abilities, such as using shortcuts are probably expected in *P. hastatus*, as this species is known to return from up to 20 km, from an unknown location to their initial roost [65]. This does not exclude the possibility that these routes are originally socially learned. Data from the late dry season and wet season of *P. hastatus* then show that bats are able to explore the landscape by using other routes when resource availability changes [48].

Despite the strong evidence that *P. hastatu*s can remember long-term high quality and abundant foraging locations which might be the most efficient strategy in the Bocas population [48], it is likely that social information transfer occurs in this population, but perhaps only during short time windows, easily missed due to short lifetime of GPS tags. Because we can only GPS track for a few days at a time, periods of resource switching after patch depletion will be hard to detect. Further we did not track young individuals, who are likely to still be in the process of learning about the distribution of resources of the landscape and who have the most gain from social learning. Finally, we did not have enough acoustic information to detect the emission of screech calls to ensure they are never used to recruit other group members to foraging trees and defend them. Once the technology advances, longer or more continuous tracking sessions which include detailed monitoring of vocalizations at different life stages will help to elucidate the reasons for group level or individual patterns.

The behavioral dynamics observed in the La Gruta bats highlights the species’ remarkable adaptability to heterogeneous landscapes. In Trinidad, female bats tend to forage together more often during the dry season, exhibiting segregation of foraging areas exploited near their roosts (~10 Km) [34,36]. This behavior is hypothesized to be driven by the necessity to defend vital resources given the landscape’s inherent scarcity [36], which may also favor foraging success [66]. Conversely, in La Gruta, females demonstrate a parallel foraging strategy by foraging and flying independently, but concentrating their foraging activities on areas that overlap spatially. Although in this study we did not measure resting associations, similar patterns as observed in the year 2016 could be occurring in 2022. Female groups might overlap spatially to form resting associations that can reinforce social ties and benefits such as predation avoidance, but still foraging independently at preferred foraging patches [39]. Remarkably, these bats fly distances exceeding twice those observed in their Trinidad counterparts, effectively meeting their daily energetic demands without, apparently, incurring higher energetic cost [39]. Further investigation about how individual variation in the use of the landscape may drive movement patterns differences between colonies would allow us to understand how such a social species is able to share feeding grounds and at the same time tolerate intraspecific competitors.

Four decades of methodological and technical advances reveals differences in how we study and interpret the behavior of a species. In Trinidad, behavioral studies relied on captures and observational methods including video and acoustic recording and counts of departing bats to understand foraging behavior (S2 Table). While these methods are valuable and do not require great financial burden, they still provide an incomplete picture of how an animal or multiple animals move in space, which could bias our understanding of observed behaviors. Currently, with the aid of biologging methods, we can follow individual movements with high-resolution location, acoustic and acceleration data and have a better approximation of possible scenarios that span individual, group and population behavioral patterns. Future research should target efforts that allow us to understand the adaptability and flexibility of behaviors of wide range social species which suffer different selection pressures given the rapid environmental changes of the local landscape.

## Data, code, and materials

Data from 2016 is available at: O’Mara MT, Dechmann DKN. 2023. Data from: Greater spear nosed bats commute long distances alone, rest together, but forage apart. Movebank Data Repository. https://doi.org/10.5441/001/1.282.

Data from 2022 is available at: Calderón-Capote MC, van Toor ML, O’Mara MT, Bayer TD, Crofoot MC, Dechmann DKN. 2024. Data from: Consistent long-distance foraging flights across years and seasons at colony level in a neotropical bat [2021–2022]. Movebank Data Repository. https://doi.org/10.5441/001/1.321

Code is available at https://github.com/mccalderonc/Intraspecific_variability_behavior_Phast.

## Supporting Information

S1 Fig Foraging times after sunset of females tracked in La Gruta colony in 2016 (n =  13) and 2022 (n =  12). Error bars (red) represent the mean and standard deviation. The whiskers represent smallest and largest values within 1.5 times the interquartile range from the first and third quartile, respectively.
(PDF)

S2 Fig Proportion of time female bats spent foraging (dark grey) and commuting (light grey) during the dry seasons of 2016 and 2022. Error bars inside the boxplots represent the mean and standard deviation. The whiskers represent smallest and largest values within 1.5 times the interquartile range from the first and third quartile, respectively.
(PDF)

S1 Table Individual information of the bats tagged and used for analysis in the present study. Measurements as follows: mass is given in grams, repro (i.e., reproductive status) of the females is nulli =  nulliparous/has never reproduced; preg =  pregnant; lac =  lactating; plac =  post-lactating. No. nights =  number of nights each bat was tracked.
(XLSX)

S2 Table General methods and outcomes from this study compare to the studies developed in the Trinidad population. DTW =  Dynamic Time Warping, AKDE =  Autocorrelated Kernel Density Estimate.
(XLSX)
