# Supplementary figures and images for: Intraspecific variability of social structure and linked foraging behavior in females of a widespread bat species (Phyllostomus hastatus)
Source: PLoS One. 2025 Mar 20;20(3):e0313782. doi: 10.1371/journal.pone.0313782 (PMC11925302; doi:10.1371/journal.pone.0313782)

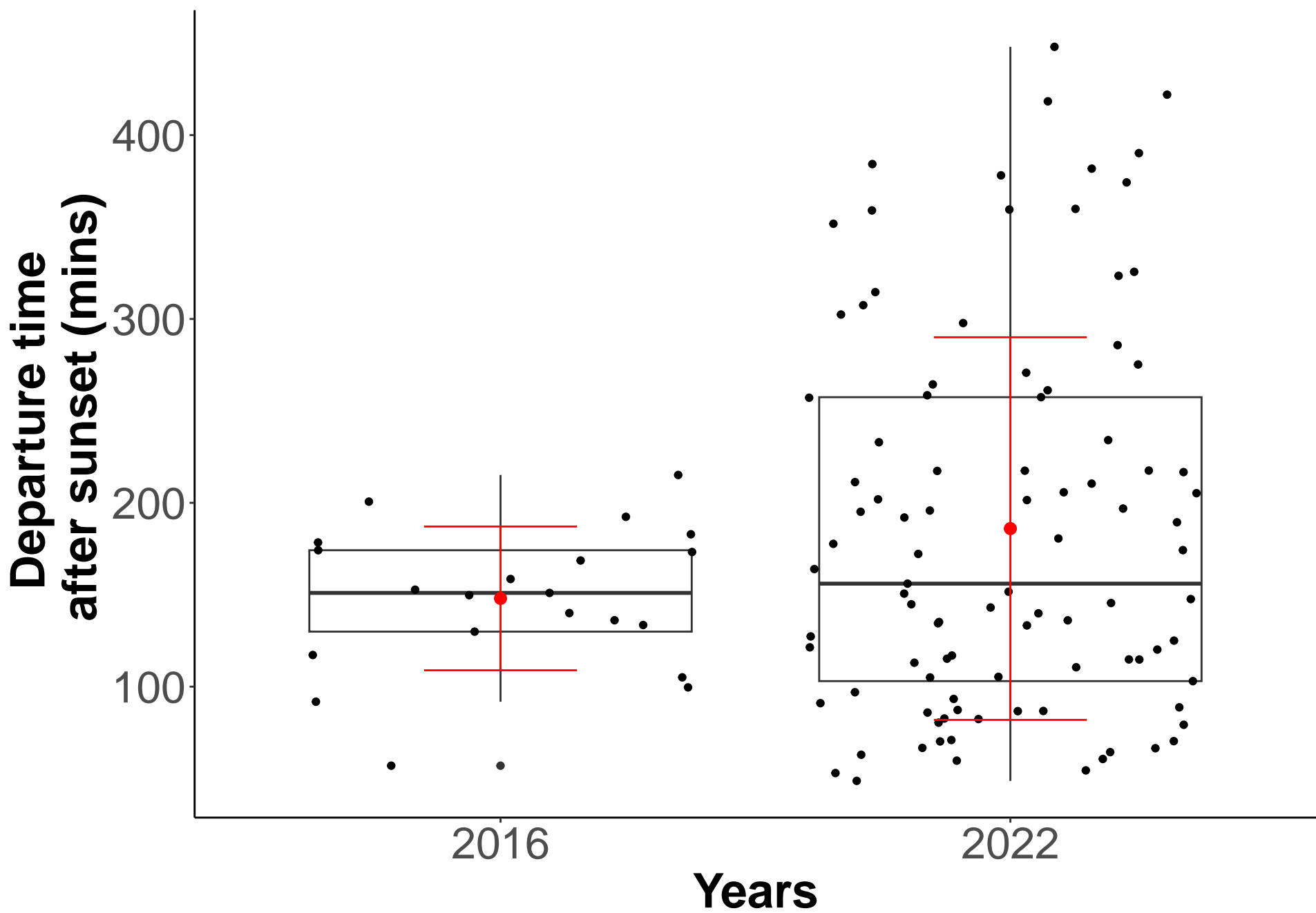

Supplement: S1 Fig — Error bars (red) represent the mean and standard deviation. The whiskers represent smallest and largest values within 1.5 times the interquartile range from the first and third quartile, respectively. (PDF) [file pone.0313782.s001.pdf]

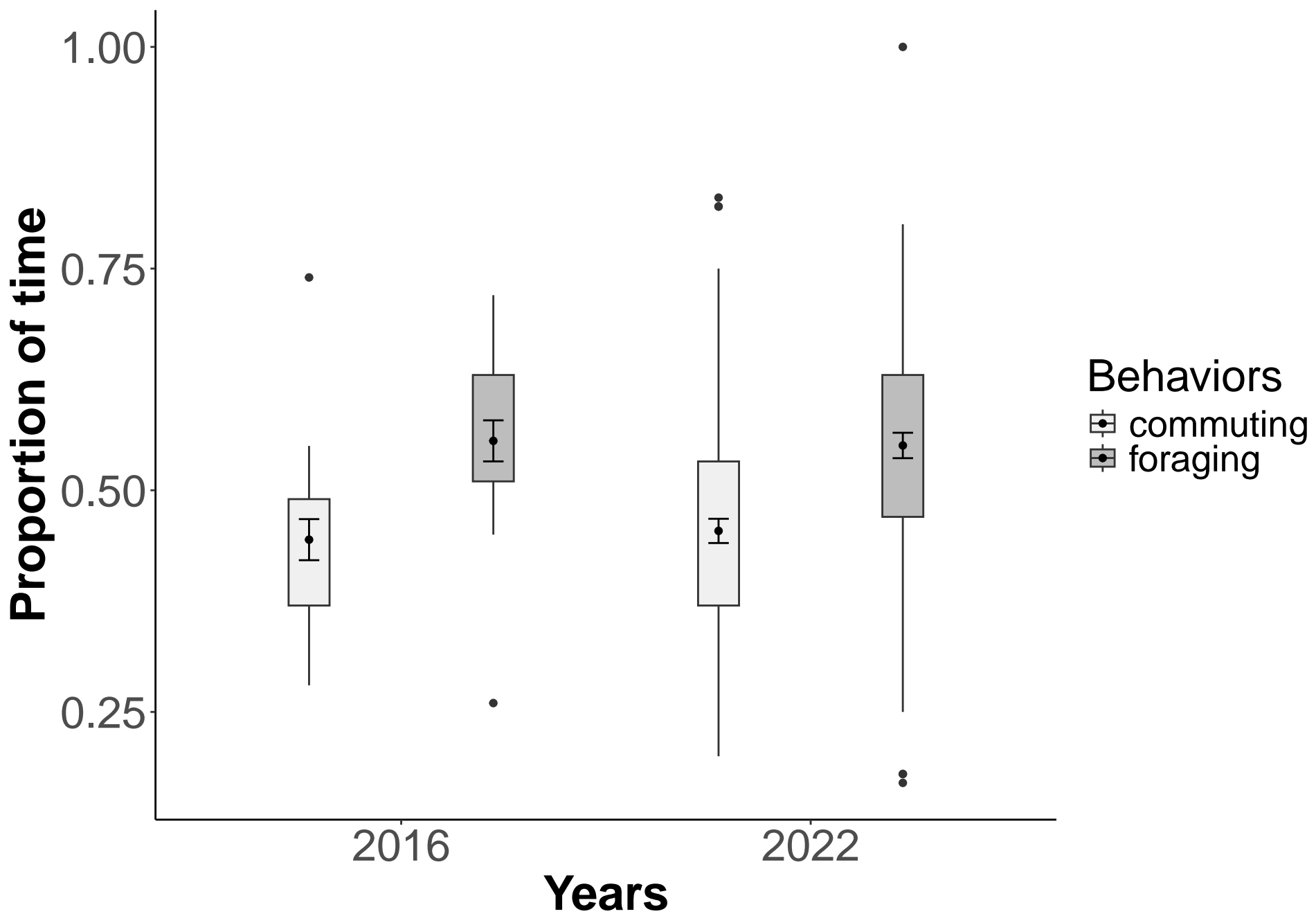

Supplement: S2 Fig — Error bars inside the boxplots represent the mean and standard deviation. The whiskers represent smallest and largest values within 1.5 times the interquartile range from the first and third quartile, respectively. (PDF) [file pone.0313782.s002.pdf]
